# Light generated bubble for microparticle propulsion

**DOI:** 10.1038/s41598-017-03114-z

**Published:** 2017-06-06

**Authors:** Ido Frenkel, Avi Niv

**Affiliations:** 1 Swiss Institute for Dryland Environmental and Energy Research, Blaustein Institutes for Desert Research, Ben-Gurion University of the Negev, Midreshet Ben-Gurion, Israel; 2 The Unit of Energy Engineering, Ben-Gurion University of the Negev, Beer-Sheva, Israel

## Abstract

Light activated motion of micron-sized particles with effective forces in the range of micro-Newtons is hereby proposed and demonstrated. Our investigation shows that this exceptional amount of force results from accumulation of light-generated heat by a micron-sized particle that translates into motion due to a phase transition in the nearby water. High-speed imagery indicates the role of bubble expansion and later collapse in this event. Comparing observations with known models reveals a dynamic behavior controlled by polytropic trapped vapor and the inertia of the surrounding liquid. The potential of the proposed approach is demonstrated by realization of disordered optical media with binary light-activated switching from opacity to high transparency.

## Introduction

Mechanical manipulation of micro and nano scaled objects are important in biology, surface science, microfluidics, and for micro-machines in general. Using light to power such manipulation is appealing due to its ability to peer into the micro and even the nano scale using microscopy. Currently, the most widespread approaches are based on radiation-pressure. Radiation-pressure has a long history starting with Kepler’s 1619 postulation as to its role in the bending tails of comets (De cometis libelli tres, page 8) and with James Clerk Maxwell showing it to be a natural consequence of his newly formed electromagnetic theory^1^. First observations were made by Peter Lebedev in 1901^2^ and soon after by Nichols and Hull in 1902^3^, with the Minkowski-Abraham controversy regarding its precise form persisting to date^4, 5^. Radiation pressure was first utilized following Ashkin’s investigation of the effect it has on micro-sized object and atoms^6^, which led, among other things^7^, to the invention of the optical tweezer^8^. Over the years optical tweezers proved a useful bridge between the macro-world we live in and micron-sized objects we wish to act upon. As such, optical tweezers found ample of use in various fields of research and technology^9^. Yet the main drawback of optical tweezers is their ability to produce no more than few pico-Newtons of force^10^. This limitation comes from the fact that radiation-pressure is based on momentum transfer^4, 11, 12^. It is clear therefore that if larger forces are needed an alternative to radiation pressure should be devised. An example of such an alternative is the phoretic motion of asymmetric Janus particles due to the absorption of light^13^. Here, however, motion results from temperature gradients rather than directly from light-generated heat. As a result, forces are not much larger than the thermal fluctuations of the surrounding liquid^13, 14^, or comparable to those found with radiation-pressure at best^15^. In the following we describe the conversion of light-generated heat into motion of micro-particle immersed in water and discuss the underlying mechanisms of such an event.

**Figure 1:**
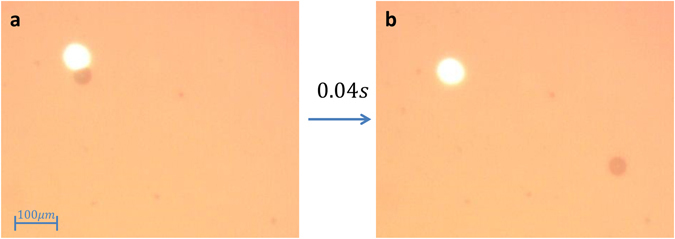
Light induced abrupt micro-sphere translation. Panel (a) shows the 42 μm diameter spherical particle and the 405 nm laser beam as the respective dark and bright patches. Panel (b) shows that 40 milliseconds later the microsphere has traversed a distance roughly 10 times its size.

Bubbles, vapor voids inside a liquid body, attract interest for well over a century due to their ubiquity and intense dynamic behaviour^16^. It is this fact combined with their efficiently in removing heat^17^ that makes them attractive for a propulsion mechanism. We hereby propose and demonstrate micro-particle propulsion with a light generated bubble. The ensuing optomechanical force (OMF) is analyzed using high speed imaging, and by comparing the observed dynamics to a known model of bubbles. Initial demonstration of the proposed approach is brought in Fig. 1 showing two sequential images out of a video capture at 25 frames per second. Panel (a) shows the bright 405 nm focused laser spot as it touches upon a micro-sphere just before motion takes place. The sample in this case is made by immersing 42 μm silver coated glass sphere in water capped between two standard cover slips. Panel (b) shows that by the sequential image 40 ms later the microsphere had traversed a distance more than 10 times its size (see the supplemented file: *video1*). The direction of the motion, in this case, is always opposite to the laser location on the micro-sphere. Estimating the effective force in this case results in tens of pico-Newtons, which is already the uppermost limit of radiation pressure based approaches (see supplementary data file for more details). This is, however, merely a lower bound. As we shall see later, a closer examination reveals a force that is *six orders of magnitude* larger than what is common for optical tweezers with the same laser power. This observation is more surprising considering that here a simple NA = 0.4 objective is used, while optical-tweezers usually take oil-immersed objectives with NA > 1. This simple demonstration merely shows the potential of the proposed approach for high-power long working distance light activated motion of micro particles.

**Figure 2:**
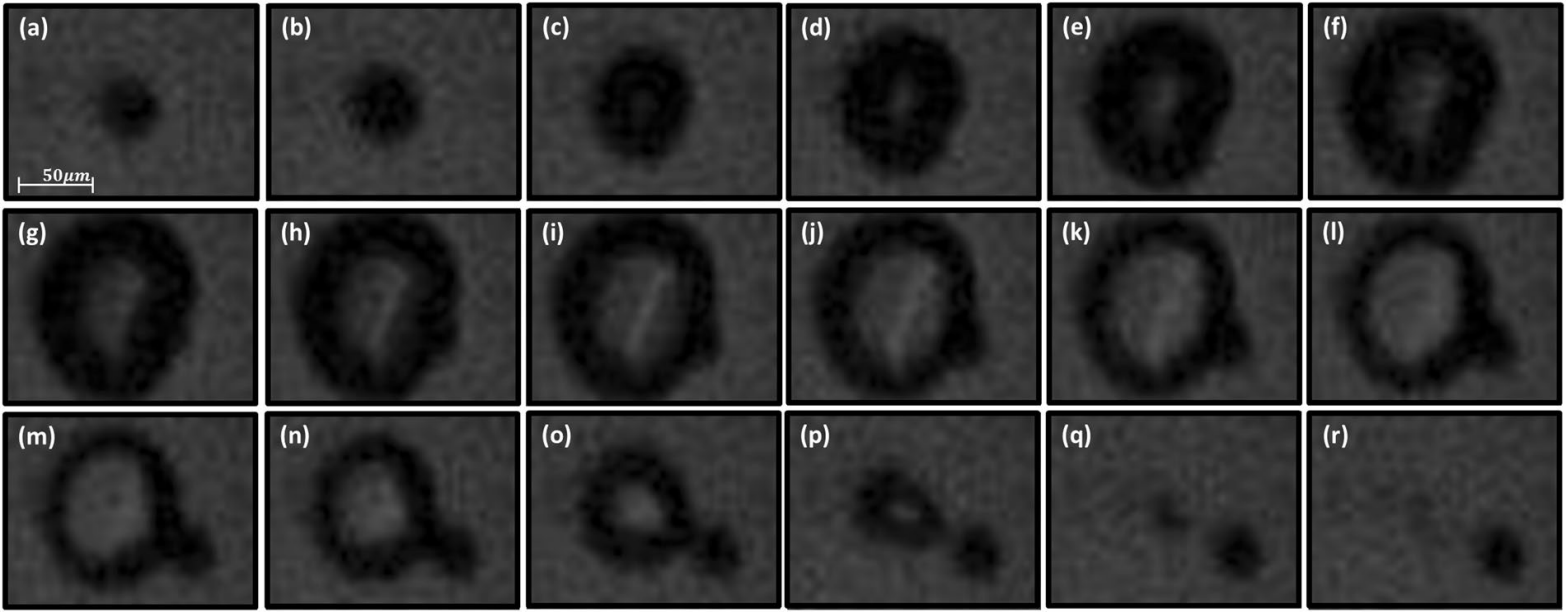
Evolution of the bubble at 2 μs time steps. Image (**a**) shows a 42 μm diameter sphere prior to the formation of the bubble. Image (**g**) shows the bubble at its maximal extent. Image (r) shows the shooting sphere after the bubble has dissolved.

A better grasp on the processes that take place during the onset of the OMF is obtained by capturing a series of images at a rate of 500,000 frames per second (see *methods* and the supplemented file: *video 2*). Figure 2 shows a consecutive set of images starting with the micro-sphere right before the bubble emerges (the 405 nm laser does not show since it is blocked by a dedicated optical filter). From there onward the expanding dark rim indicates the progression of the vapor/liquid interface of the bubble. The micro-sphere is not observed at these early instances since it is carried along with the bubble interface that appears as the dark rim. The offset of the micro-sphere from its original location terminates the absorption of laser light by the micro-sphere and thus shuts-off the power supply for the expansion of the bubble - The process is therefore self-controlled. The bright region at the center of the bubble, first appearing at panel (d), shows that the vapor has reached the glass slides. As a result, from that point onward, the bubble becomes more of a disc than a sphere (excluding the final stages of collapse). We have also performed tests with larger separation between the glass slides that were expected to yield more spherical bubbles. These displayed similar dynamics but with less crisp imagery and are therefore not shown here. The next two images in panels (e) and (f) show that the bubble develops a dent as it expands. This dent is caused by the micro-sphere whose progression lags behind the advancing front of the bubble. The micro-sphere eventually catches-up and begins to perturb starting at panel (g). Panels (g) and (h) show the bubble at its maximal extent, from there onward the bubble collapses while the micro-sphere continues at its original outward trajectory. This causes the bubble to develop a bulge rather than the former dent, as seen in panel (m) for example, resembling somewhat the pinch-off effect^18^. Finally, as seen in panel (p), the micro-sphere separates from the bubble and from there onward the micro-sphere is freely moving in water while the bubble continues to dissolve until it completely vanished. Although no two events were alike, similar trends were found in hundreds of instances and with different sizes of micro-spheres.

**Figure 3:**
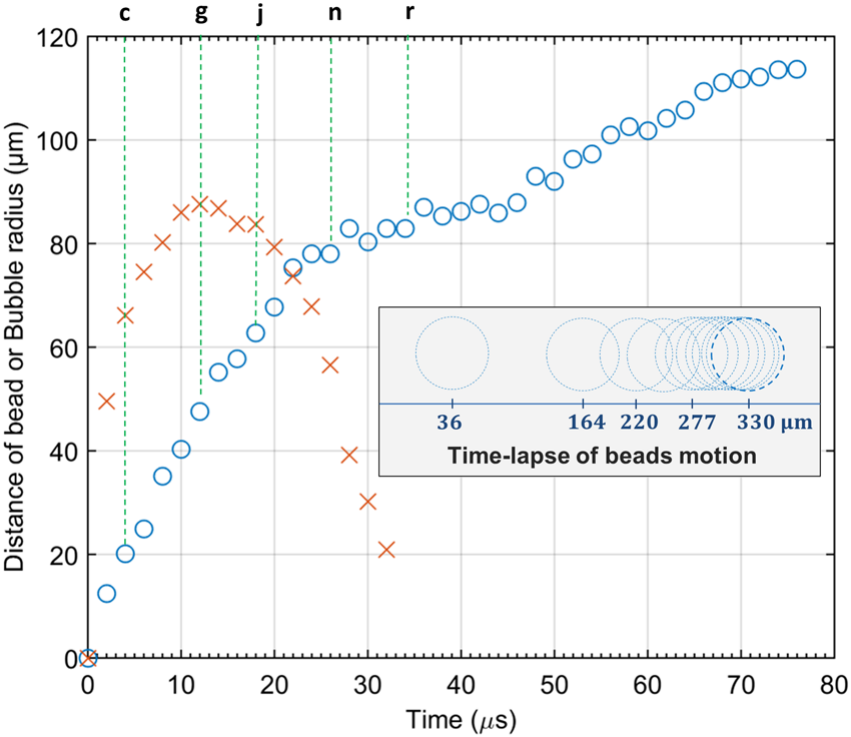
Typical micro-sphere motion and the corresponding bubble radius. Micro-sphere location is shown with blue ‘o’ marks while the radius of the bubble with orange ‘x’ marks. Letters corresponds to a respective frame in Fig. 2. Inset depicts the motion of a typical micro-sphere after the bubble has vanished with 200 μs between consecutive locations.

Figure 3 shows with blue ‘o’ marks the micro-sphere location as a function of time, while letters at the upper horizontal axis indicates the corresponding panels of Fig. 2. For comparison, the effective bubble radius $\tilde{R}$ versus time is shown in orange ‘x’ marks. This radius is found from $\mathop{\,R}\limits^{ \sim }=\sqrt{S/\pi }$ with *S* the observed bubble surface, a definition that mitigates somewhat the effect of deformation at the later stages of the bubble motion. Few things comes to mind: First, the expansion stage of the bubble, from initiation and until the turning point at 12 μs, is mirrored as the bubble collapses starting from the turning point and ending by approximately 26 μs. This symmetry is a clear indication of small dissipation of energy from the bubble to its surrounding. This is not surprising considering the major dissipation mechanisms of bubbles: mass and heat diffusion^19–21^, the viscosity of water, and acoustic radiation (the emission of shock waves)^21, 22^. Typical time scales for evaporation is milliseconds^19, 20^ and the characteristic time for thermal diffusivity is ${t}_{T}={\tilde{R}}^{2}/{\alpha }_{g}\approx 100\,\mu s$ (for vapor thermal diffusivity *α*_*g*_ = 2.338 × 10^−5^ *m* · *s* ^−2^ and typical bubble radius $\tilde{R}=100\,\mu m$). Therefore, mass and heat diffusion does not affect the dynamics since their time scales are larger than the typical pulsation time of the bubble, which is ~ 40 μs in our case. Dissipation due to the viscosity of water and the emission of shock waves are also negligible throughout the *observed* part of the bubble since the velocity of the vapor/liquid interface at these stages, as seen from Fig. 3, does not exceed 5 *m* · *s* ^−1^. Intense evaporations or condensations are however expected at the very early and last stages of the pulsation that are not observed here. Indirect evidences for the emission of shock waves are however found and would be discussed later. Another source of dissipation, which is particular to our case, is the friction of the micro-sphere with the surrounding water. This mechanism is however not significant during the combined bubble micro-sphere motion since the radial velocity field in the liquid surrounding the bubble is $v=\dot{R}{R}^{2}/\,{r}^{2}\,$where *R* is the bubble radius, and *r* ≥ *R* is the radial coordinate^20^. The bubble therefore pushes the water as it expands so friction is minimal as long as the micro-sphere goes along with the bubble. This, however, changes once the micro-sphere perturbs at the later stages of the combined micro-sphere/bubble motion and even more so after separation.

Most important, however, is that the solid surface upon which the bubble is formed, which is the micro-sphere, is moving in respond to the forces from bubble. The salient effect of this motion is to terminate the energy source for the bubble expansion by removing the micro-sphere from the focused light source. This motion causes the dent in the bubble starting at panel (e) of Fig. 2 and the later bulge. It also causes the slowing down of the micro-sphere starting 24 μs after bubble initiation. Figure 2 shows that by this time the micro-sphere is significantly perturbing beyond a highly deformed bubble. Two mechanisms are therefore responsible for the slowing down of the micro-sphere: Friction with the surrounding water that was discussed before and the surface tension forces from the receding bubble. A larger scope of a micro-sphere’s motion is brought at the inset in Fig. 3, time between two depicted locations is 200 μs. This depiction starts right after the bubble has vanished where the micro-sphere has traversed 36 μm from its initial location and up until 330 μm where two consecutive locations could no longer be distinguished. The micro-sphere reached a complete halt at 350 μm from its initial location. The observed deceleration is a direct outcome of the micro-spheres friction with its surrounding water as shown in the supplementary data file.

**Figure 4:**
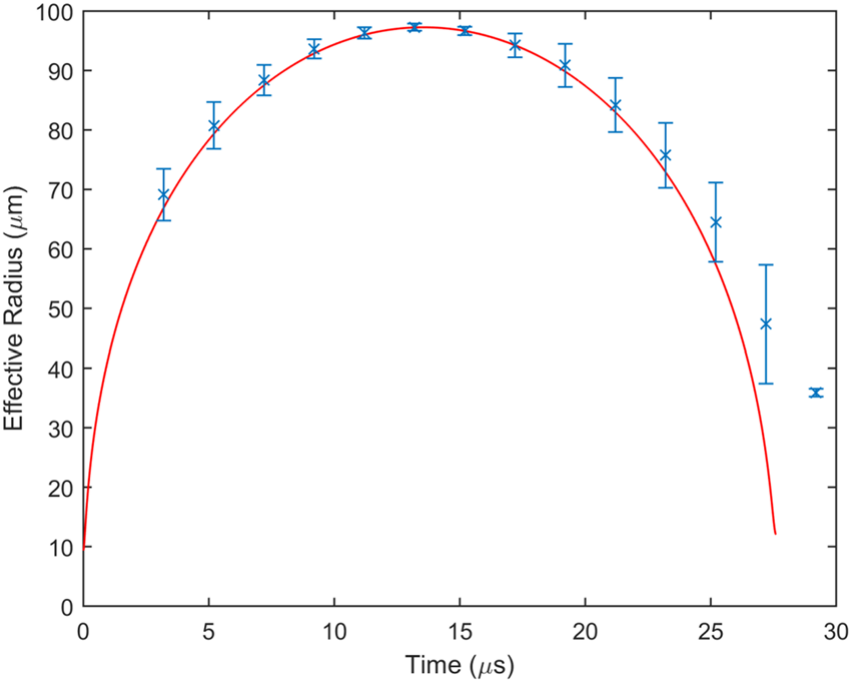
Evolution of the effective bubble radius with time. Blue ‘x’ marks shows average bubble radius with error bars depicting the standard deviation in observation. The expected dynamics from an inertially controlled spherical bubble, following Eq. (1), is shown in red.

To gain better insight as to the processes that take place during the combined bubble-micro-sphere dynamics observations were matched to known model of bubble dynamics. In order to do so tests were performed while maintaining 90 μm between the cupping glass windows, which allows more spherical bubbles to develop. Figure 4 shows the time evolution of the average bubble radius for 7 OMF occurrences (more details in the supplementary data). This trend is compared to the known dynamics of spherical bubbles as given by the Rayleigh-Plesset equation^19–21^:

1 $${P}_{V}(t)-{P}_{\infty }-{P}_{S}=\rho \,[R(t)\ddot{R}(t)+\frac{3}{2}\dot{R}(t)].$$

Here *R*(*t*) is the instantaneous bubble radius, *P*_∞_ is the pressure of liquid far away from the bubble, *ρ* is the density of water, and overdot represent time derivative. The effect of viscosity is neglected since it found play only a minor role in our case. The inertial behavior of the bubble emerges once adopting the polytropic model for the vapor inside the bubble^20, 21^:

2 $${P}_{V}(t)={P}_{0}{(\frac{{R}_{0}}{R(t)})}^{3\gamma },$$

with *P*_0_ and *R*_0_ are the initial pressure and bubble radius, respectively, and *γ* is the polytropic constant. Finally, the pressure due to surface tension is given by:

3 $${P}_{S}(t)=\frac{2\sigma }{R(t)},$$

with *σ* as the surface tension coefficient of water. Equations (1), with insertion of (2) and (3), are integrated numerically to produce the red line in Fig. 4. The initial pressure was taken to be at the critical point of water, i.e. *P*_0_ = 22.064 MPa, while adiabatic process was considered by taking *γ* = 1.33 (considering isothermal condition by choosing *γ* = 1 did not change the results significantly). Also, the density and surface tension coefficient were taken at their standard values of *ρ* = 1000 kg · m^−3^ and *σ* = 0.07 kg · s^−2^, respectively. The best fit emerged for ambient pressure *P*_∞_ = 44 kPa and initial radius *R*_*i*_ = 9.35 *μm*. Agreement of the model with measured data is surprisingly good considering the effect of the micro-sphere on the bubble and the fact that, albeit a larger spacing, the relative proximity of the glass window is expected to flatten the bubble somewhat. The agreement may be aided, however, by the fact that the Rayleigh-Plesset model for 2D bubbles is essentially the same as that of 3D^23^. Another important point is that here a polytropic behavior was assumed for the vapor and not, as it is usually done, to a contaminate gas. This choice was motivated by the fact that the bubbles vanished within 50 μs or less, which was attributed to rapid condensation rather than the much slower contaminant diffusion^20, 21^. None the less in some rare occasions a rebound bubble, much smaller in size and pulsation time, has been observed.

Beyond the good agreement Fig. 4 shows that deviation exists between model and observation at the early stages of bubble and more so at the later stages of its collapse. This is due to the non-adiabatic process that takes place at the early and final stages in the form of rapid evaporation or condensation, respectively. The time scale of such processes should be in the order of the pressure equalization time $\,{{\text{t}}}_{{\rm{P}}}={\mathop{{\text{R}}}\limits^{ \sim }/c}_{{\rm{g}}}\approx 0.2\,\mu {\rm{m}}$ in our case (for $\mathop{{\text{R}}}\limits^{ \sim }=100\,\mu {\rm{m}}$ bubble and vapor speed of sound *c*_g_ ~ 477.5 m/s), which is clearly below our 2 μs temporal resolution. Also, due to the large velocities at these extremes, the emission of shock waves is likely to occur - an assumption supported by the faint but still audible ‘click’ sound that could have been heard in the lab at the initiation of an OMF event.

Based on these observations we propose the following sequence of events to take place at the onset of an OMF event: First, the surface of the micro-sphere is heated by absorbing laser light. The duration of this heating period varies from a few seconds at low laser power, and down to few milliseconds at full power of 140 mW. This time scale is also confirmed by a direct measurement of the heating time and by the finite-element simulation brought in the supplementary data file. The micro-sphere at this stage acts as reservoir for the energy of light in the form of heat. This heating continues until a film of nearby water makes a phase transition to become a high pressure vapor. The good agreement with the model indicates that the temperature of water at this stage is close its critical value - a situation somewhat resembling steam-explosion^24^ only at much smaller scales. This phase-transition is likely to take place within the pressure equalization time, which is 0.2 μs in our case. Deviation of the observed dynamics from the model at the early stages of the bubble expansion is the surplus of this embryonic highly non-adiabatic event. As the bubble expands the vapor cools and its pressure drops giving rise to almost dissipation-less dynamic behavior, as indicated by the symmetry of the bubbles inflation and deflation stages. This is also where the bubble is first captured on camera. Since the micro-sphere is carried along with the bubble, it is removed from the laser so the energy source for the bubble inflation is terminated. The ensuing slowing down of the bubble, as seen in Figs 3 and 4, indicates that vapor pressure *P*_V_ has fallen below the combined effect of *P*_∞_ and *P*_S_. This observation is also in agreement with the calculated pressure plots brought in the supplementary data. Once the inertia of the bubble is exhausted the bubble begins to collapse but still in an adiabatic manner. The bubble is not perfectly spherical as it collapses since most of it recedes while the micro-sphere drags along the close-by parts due to tension along the three-phase contact line^17^. Under these opposite trends the bubble develops a significant deformation and the micro-sphere slows down until complete separation is obtained. The micro-sphere is now freely moving in water as the bubble undergoes its final non-adiabatic stages of condensation. The small acceleration that the micro-sphere experiences at 44 μs in Fig. 3 indicates that the bubble/micro-sphere interaction is not over even after the bubble is apparently gone. This is a second indication for the emission of shock-waves (the first being the ‘click’ sound). It is important to note that this final acceleration stage was a typical occurrence and not particular to a specific OMF event.

Although the proposed OMF shares some similarity with radiation-pressure based approaches, mainly being light-activated, some fundamental differences do exist: Being based on momentum transfer, radiation-pressure is instantaneous and directly proportional to the intensity of light. The above mentioned OMF, on the other hand, comprises a chain of events (Absorption of light by the micro-sphere → Heat accumulation and conduction to water → Phase change in water → rapid bubble expansion and collapse → kinetic energy transfer to the micro-sphere) that are neither instantaneous nor linear. For example, below a critical power of light the rate of heat-removal from the micro-sphere to its surroundings is sufficient to prevent the occurrence of the phase transition in the nearby water and therefore prevents the OMF from ever to occur. Above that critical power, the occurrence of an OMF is certain but the period of heating before such event occurs may vary. The intensity of the OMF, however, showed little dependence of the power of the light source as long as it was above the critical activation level. For high enough laser power of about 100 mW in our case the onset of OMF was achieved with typical heating period of a few milliseconds. Albeit these obvious differences we attempt to compare the OMF with radiation pressure by assigning the OMF an equivalent force, which is the force that would accelerate the micro-sphere to a given speed at a given time. From Fig. 3 we find that 40 μs after initiation, the micro-sphere is freely moving in water at a velocity *V* = 0.75 m/s. Considering 40 μm diameter micro-sphere that is made almost entirely from glass (density 2.5 gr · cm^−3^), its mass *M* is 84 × 10^−12^ kg. The linear momentum of the micro-sphere at this stage of its motion is therefore *P* = *M* · *V* = 63 · 10^−12^ kg · m · s^−1^. From $\,P=\int Fdt\approx \tilde{F}{\rm{\Delta }}t$, and with a time scale of Δ*t* = 40 μs, an effective force of $\,\tilde{F}\approx 1.6\cdot {10}^{-6}\,{\rm{N}}$ emerges. With this figure in mind, we now estimate the upper limit of the radiation-pressure force from the same light. The OMF was obtained from a 100 mW power laser beam with wavelength λ = 405 nm. Since each photon in this case carries $\hslash \omega \approx 5\cdot {10}^{-19}$ Joule of energy (*ω* = *c*/*λ*) the photons rate is *R* = 2 · 10^17^ s^−1^. Since the momentum of a single photon is $p=\hslash k=1.6\cdot {10}^{-27}$ Kg · m · s^−1^ (*k* = 2*π*/*λ*) the force from fully backscattering this flux is *F* = 2 × *p* × *R* ≈ 6.7 × 10^−10^ N. The OMF was therefore experimentally demonstrated to be four orders of magnitude larger then uppermost theoretical value of radiation-pressure for the same light intensity. In practice, focusing of the beam and spherical shape of the micro-sphere results in much smaller radiation-pressure forces - Optical tweezers, for example, are known to delivers no more than few pico-Newtons at similar laser power and with tighter focusing of the beam^10^. It is seen therefore that changing the paradigm from momentum-transfer to energy-transfer enable forces that are up to six orders of magnitude larger than what was achieved so far.

**Figure 5:**
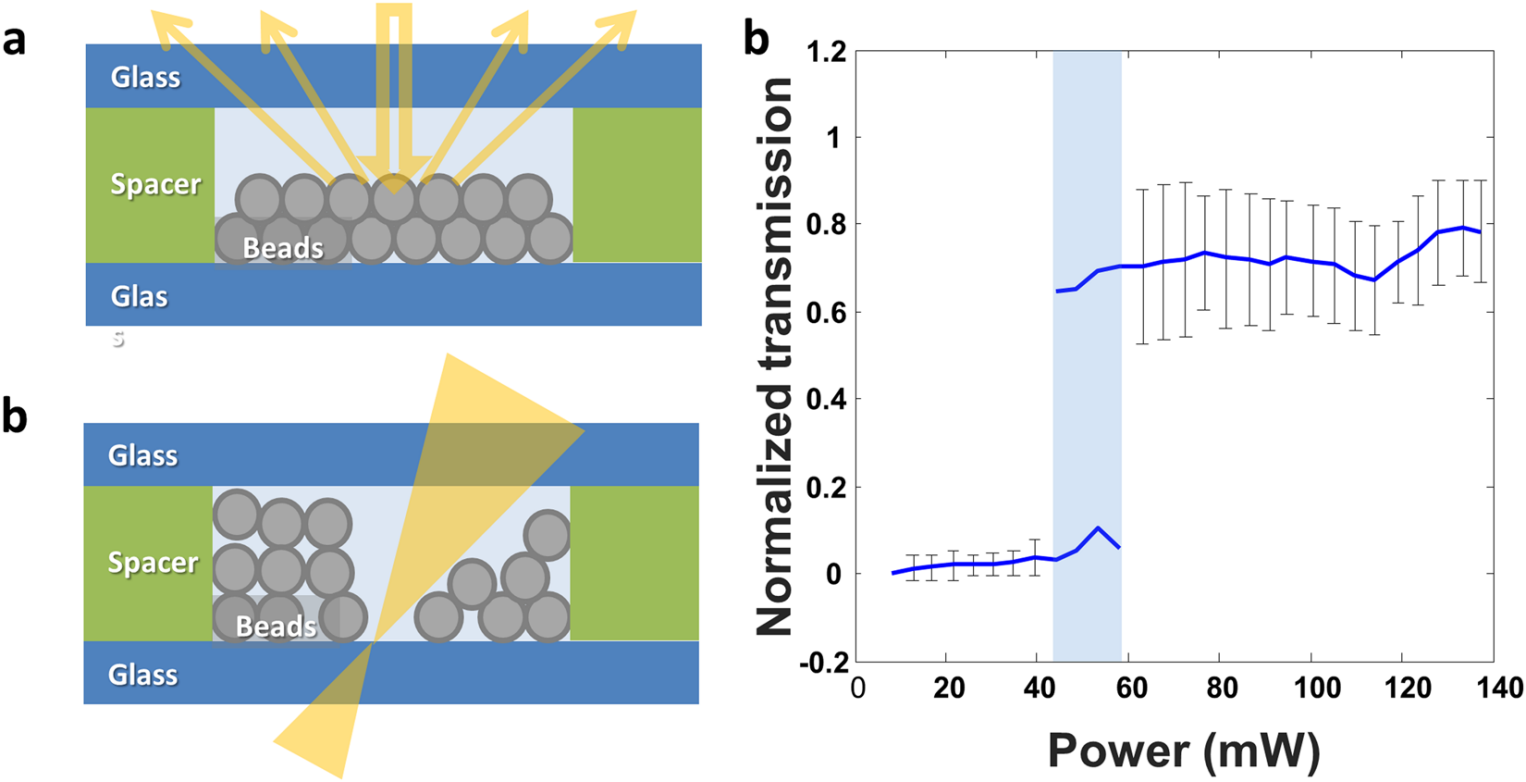
Disordered reactive optical element. (**a**) Dense collection of microparticles blocks transmission by reflecting and absorbing the incoming light at mild to low intensities. (**b**) Above a critical power level of the incoming light, the OMF removes the particles out of the beam path such that transparency emerges. (**c**) Transmission of the disordered optical substance as a function of incoming laser power. The critical power beyond which transparency arises is marked in blue.

Disordered optical media attracted recent interest from a fundamental and an applied point of views^25, 26^. We, therefore, chose to demonstrate the proposed OMF by forming a substance with binary light-activated switching from opacity to transparency. The proposed device is made by loading microparticles into the sample until a dense collection is formed. This dens collection blocks the transmission of light due to the reflective/absorptive nature of the micro-spheres as shown schematically in Fig. 5(a). When light is focused onto this substance the OMF repels the micro-spheres from that region such that transparency emerges, as shown in Fig. 5(b). The measured transmission of this substance at varying laser intensities is shown in Fig. 5(c) with critical power beyond which transmission emerges is highlighted in blue. This shows the potential of the proposed approach to form optical materials for applications ranging from optical processing to imaging and displays. A video showing the micro-spheres as they repel and rearrange around a moving laser spot is given in the supplemented file: *video3*. The ability of particles that are resting against each other to reorganize is a clear indication of the large forces the proposed OMF produces.

In conclusion we demonstrated the conversion of light’s energy to a kinetic motion of micron-sized particles that are immersed in water. The proposed approach is based on the accumulation of heat combined with the efficiency of phase-transition in transporting and converting heat to other forms of energy. As a result micron sized object were propelled at unprecedented speed close to one meters-per-second, or equivalently were acted upon with forces of micro-Newtons, while maintaining control of the direction of motion. Both of these merits are six orders of magnitude larger than what is common at present devices. Despite the dependence on the dynamics of a bubble, no trace of the vapor remains few tens of micro-seconds after initiation. Our analysis indicates that, with the exception of few microseconds at the early and last stages, the process is dissipation-less. The opportunities that this powerful tool offers are demonstrated with the realization of a disordered optical substance that has the ability for light-activated binary switching from opacity to transparency.

## Methods

### Sample preparation

The sample was made from glass microspheres that are coated with a thin (200 nm) layer of silver (SLGMS-AG-2.5 from Cospheric, USA). The diameter of a typical sphere (micro-sphere) that was examined varied between 40 μm to 60 μm. The micro-spheres came as dry substance that was immersed in distilled water and placed between two glass cover slides. Proper separation between slides was maintained with a 90 μm spacer. Separation was required to allow undisturbed bubble dynamics and free motion of the micro-spheres. More details are given in the supplementary data file.

### High frame rate imaging

Different cameras were used throughout the experiments. Figure 1 was captured with a standard CMOS camera (DCC1545M from Thorlabs, USA). The images in Fig. 2 were taken with a high speed camera Phantom v12.1 from Vison Research USA. Each frame comprised of 128 × 32 pixels which allowed for a rate of 500,000 frames per second at exposure of 1.8 μs (2 μs between each frame). The images shown in Fig. 2 were acquired without the spacer between slides for a more crisp view of the spheres motion that was somewhat suppressed in-turn. See supplementary data for more details.

### Simulation

Numerical calculations were obtained using Ronge-Kutta method up to the fourth order (error of order five).

## Electronic supplementary material

Supplementary material
