# Supplementary material for: Light generated bubble for microparticle propulsion
Source: Sci Rep. 2017 Jun 6;7:2814. doi: 10.1038/s41598-017-03114-z (PMC5460221; doi:10.1038/s41598-017-03114-z)
Supplement: Supplementary file 1 — Supplementary material [file 41598_2017_3114_MOESM1_ESM.pdf]

Supplementary material for “Light generated bubble for microparticle propulsion” by Ido Frenkel[a,b] and Avi Niv*[a]

(a) Swiss institute for Dryland Environmental and Energy Research, Blaustein Institutes for Desert Research, Ben-Gurion University of the Negev, Israel.

(b) The Unit of Energy Engineering, Ben-Gurion University of the Negev, Israel

* aviniv@bgu.ac.il

Experimental setup and sample preparation

A schematic depiction of the experimental setup is shown in Fig.S1 Illumination from a $405\mathrm{nm}$ diode laser (L) with variable power of 0-140 mW is focused to the sample by Newport m-20X 0.4NA objective (O1), and the transmission from the sample is collected by a second Leica achro 4/0.1 objective (O2). Two beam splitters (BS1, BS2) are used to direct transmitted light to a power meter (PM), when used, and for sample illumination (IL) for the camera (C). Different cameras were used throughout the experiments; Fig. 1 was captured with a standard CMOS camera (DCC1645C from Thorlabs, USA), whereas the images in Fig. 2 were taken with a high speed Phantom v1211 camera from Vison Research, USA. A longpass filter Thorlabs FEL0500 (LP) was used to block the laser radiation from reaching the camera. The sample, whose enlarge schematic depiction is also shown in the figure, is nominally composed from $43-62\ \mu\mathrm{m}$ diameter glass microspheres coated with $200\ \mathrm{nm}$ of silver from Cospheric, USA (part number SLGMS-AG-2.5). The microspheres come as a dry substance that was immersed in distilled water and placed between two glass cover slides. When separation between slides was required, as for example in the experiments leading to Fig. 3, a $90\ \mu\mathrm{m}$ plastic spacer was inserted between the two glass slides. For the high speed video measurements, each frame comprised of 128×32 pixels that allowed capturing 500,000 frames per second, which is $2\ \mu\mathrm{s}$ between consecutive frames, at exposure of $1.8\ \mu\mathrm{s}$. The images shown in Fig. 2 were acquired without the spacer between slides since a clearer image was obtained in this way. The experiments leading to Fig. 4 were made while maintaining separation that allowed the bubble to develop a more spherical shape.

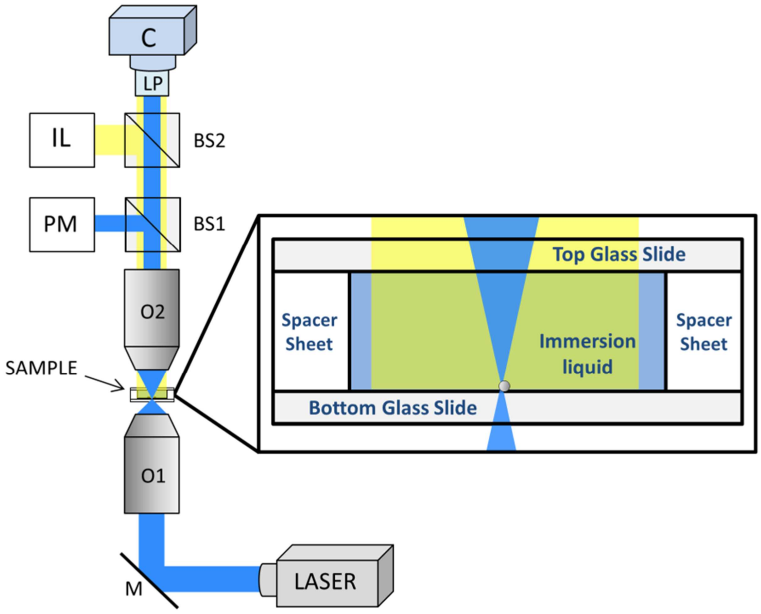

Figure S1 | Schematic depiction of the upright optical setup. Illumination form a light source (L) is focused onto the sample by a 45° mirror (M) and an objective 20X (O1). A second 40X objective (O2) collects light for imaging and transmitted power measurements. Two beam splitters (BS1, BS2) are used to direct transmitted light to a power meter (PM), and light from the illuminator (IL) to the sample. Imaging is obtained with a camera (C). A longpass filter (LP) is used to block the undesired laser light from reaching the camera. Enlarged schematic depiction of the sample layout is also given.

Estimating the equivalent force

Fig. 1 shows OMF event captured with a regular camera at a rate of 25 frames per second. Knowing the distance the microsphere covered and having an upper bound for the duration of that this translation occurred, a lower bound on the force is obtained from $m\Delta x/\Delta t^2$, where Δx and Δt and are distance and duration of motion and $m = (4\pi/3)\rho r^3$ is the mass of the microsphere. Also, the density and radius of the microsphere are given by ρ and r, respectively. In our case $r = 20$ μm microsphere made almost exclusively from glass, so $\rho = 2500$ kg/m^3. From Fig. 1 the beam translated roughly a distance of 400 μm at time no longer than 0.04 s and therefore the lower bound of the force in this case is 21 pN.

Bubble radius and microsphere location

Fig. 3 shows the bubble radius and the location of the microsphere. These were obtained by allocating the desired properties on the film-strip whose first 18 frames are shown in Fig. 2. A more graphical laden version of Fig. 2 is shown in Fig.S2: The green cross indicate the original location of the microsphere that serves as a reference for the later motion of the microsphere and the displacement of the bubble as it

expands, collapse, and later vanishes. The yellow circle with the respective cross at its center shows the location of the microsphere at different instances. Finally, the bubble is highlighted with a white circle whose center is indicated by the white cross. It is interesting to note that while the microsphere translates a significantly during the process, the bubble moves only slightly in the opposite direction.

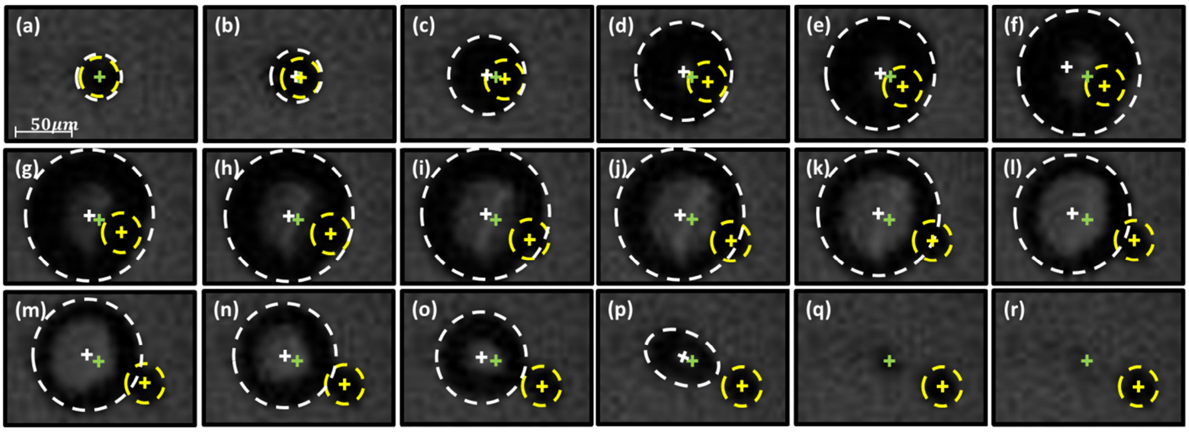

Figure S2 | Bubble radius and microsphere location at different instances after the bubble was first detected. Time between consecutive frames is 2 μs. The Green cross indicates the original location of the microsphere. The yellow circle with the respective cross at its center shows the location of the microsphere at different instances. The white circle with the corresponding white cross indicates the bubble and its center respectively.

A Larger scope of the microsphere's motion

Measurements of the microspheres location after the bubble fully condensates were made with the high speed camera at 500,000 frames per second and shown in Fig.S3. The blue dots in Fig.S3 show location of the microsphere as a function of time, these are also the measurements that gave rise to the inset in Fig. 3. This behavior corresponds to movement of an object in a liquid, with friction force proportional to the object velocity:

$$m\ddot{x} = -bv, \tag{S1}$$

where x and v are the location and velocity of the microsphere and the double overdot represent the time second derivative of the microspheres location. $b = 6\pi\eta a$ is the drag force coefficient of a sphere in accordance to Stokes law, where η is viscosity of water and a is the radius of the microsphere. The solution of Eq. (S1) is:

$$x = v_0 \frac{m}{b}\left(1 - e^{-\frac{b}{m}t}\right), \tag{S2}$$

where v_0 is the initial velocity (from the moment the bubble vanished), m is the mass and t is the time. The red line in Fig.S3 shows the trend of Eq. (S1) with $v_0 = 0.6\ m/s$ and room temperature viscosity of water, $\eta = 8.94 \cdot 10^{-4} \frac{kg}{s \cdot m}$. The small disagreement the calculated motion from the measured one could result from the confinement of the two cover slides as well as from a certain amount of spin that the microsphere obtains.

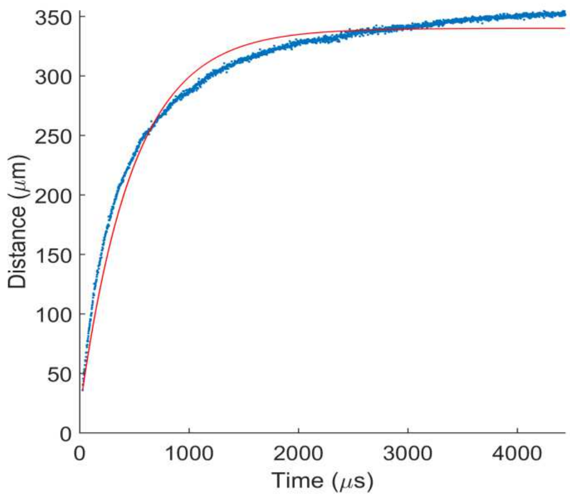

Figure S3 | A larger scope of the microspheres motion. The figure shows a larger scope of the microspheres motion after the bubble has vanished. Blue dots represent the experimental measurements and the red line is the corresponding calculated motion if a spherical object in water.

Statistics of bubble expansion

Variation between individual events makes it hard to facilitate a useful comparison of the measured results with the Rayleigh-Plesset model of the dynamics behavior of a spherical bubble. Variations in this case arise from the explosive nature of the phase transition and its sensitivity to imperfection on the surface of the microsphere and the surrounding water. Since our setup would not allow us to repeatedly excite a given microsphere at the exact same location time after time, we have reputedly excited a microsphere and from the dispersion of outcomes we isolated a representative subset that presumably represents similar events. Accordingly, we have chosen two parameters for quantification of a possible outcomes; the maximal radius of the bubble and the duration from initiation until it dissolves back to liquid water. A histogram of 53 OMF events of the same microsphere that are ordered along these two parameters is shown in Fig.S4. The diagonal arrangement indicates correlation between these two merits of the bubble, which is after all expected. Also, the results are approaching a normal

distribution that is centered at maximal radius between 96 to 98 μm and duration of 26 to 28 μs. We therefore chose this most probable outcome as our representative subset of events for the analysis - this is the 7 bubble set whose mean radius and deviation are portrayed in Fig. 4.

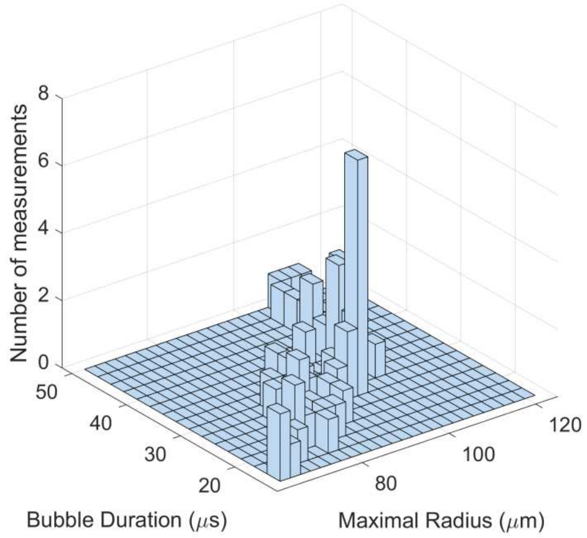

Figure S4 | Statistics of bubble expansion. Maximal effective radius and time total pulsation time for 53 different occurrences with the same microsphere. The results are approaching a normal distribution that is centered at maximal radius between 96 to 98 μm and duration of 26 to 28 μs.

Duration of heating period and simulation of temperature distribution

Measurements of the heating time were made by capturing a sequence of frames using Ximea MQ003MG-CM camera pushed to 2398 frames per second while heating the microsphere with 140 mW laser until motion was detected. The laser was triggered with a mechanical shutter SHB05 from Thorlabs, USA. These measurements indicated a typical heating time of 3 ms. This heating period is also confirmed from a finite element simulation (COMSOL) of the temperature buildup with time of laser heated microsphere. Simulation parameters were chosen to reproduce as much as possible the realistic conditions: 50 μm glass microsphere covered by 200 nm silver were considered, a 405 nm laser at a power 100 mW with beam waist of 20 μm. A cross section of the temperature distribution on the microsphere and its surroundings after 3ms of heating is shown in Fig.S5. The beam was focused 10 μm to the right of the south pole of the microsphere. Few things are notable in this simulation: First, the water close to the heat source had reached the critical temperature of 650 K by these 3 ms of heating - in agreement with the measurement of the heating period described above. Secondly, simulation shows

that the heat is not evenly distributed around the microsphere by that time, in agreement with observation that the bubble always occur where the laser was heating the microsphere.

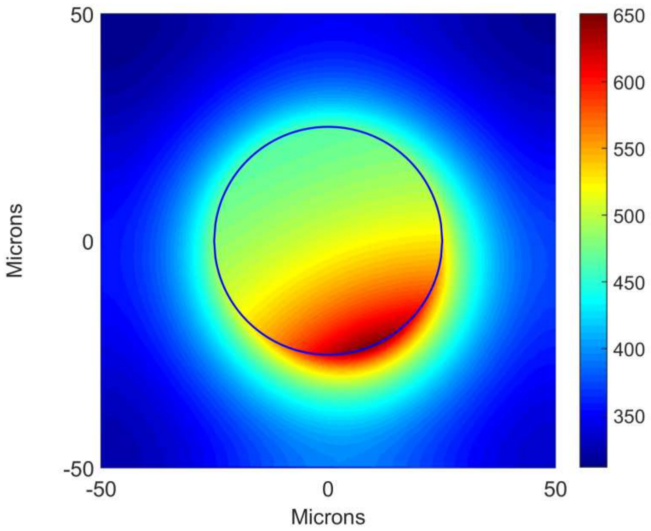

Figure S5 | Simulation of the temperature distribution on the microsphere. Temperature across a 50 μm glass sphere covered with 200 nm of silver after 3ms of heating. Heat is supplied by optical absorption of a 100mW light beam with 10μm waist that is centered 10μm to the right of the symmetry axis of the sphere. False color indicates temperature in Kelvin.

The pressure difference

The expected pressure difference was obtained from the bubble dynamic calculation described in the manuscript, the result is given in Fig.S6. Figure S6 shows the pressure difference simulation for the adiabatic period of the bubble. Not shown in the figure is the initial large pressure difference of $P = 22.064$ MPa that corresponds to the phase transition that took place. The figure in turn focuses on the region of moderate pressure, where adiabatic behavior takes place. The rapid expansion of the bubble causes the pressure to drop until a negative value is established starting at about 1 μs after initiation. It is clear, therefore, that by the time the bubble is first observed, after about 2 μs, it is already when its rate expansion is slowing down. As the bubble keep on to expand, the pressure continues to drop unit it attains it minimal value of -44 kPa after 13 μs. This is also the point where the bubble velocity is zero and expansion turns to collapse. The bubble speeds up its collapse until the pressure retains a positive value once more. Under the surface tension forces the bubble continues to collapse and the pressure rises accordingly (also not shown in the figure). Finally, with the lack of a heat

source, since the microsphere is already shifted from the beam, condensation kicks-in and the bubble dissolves back to liquid.

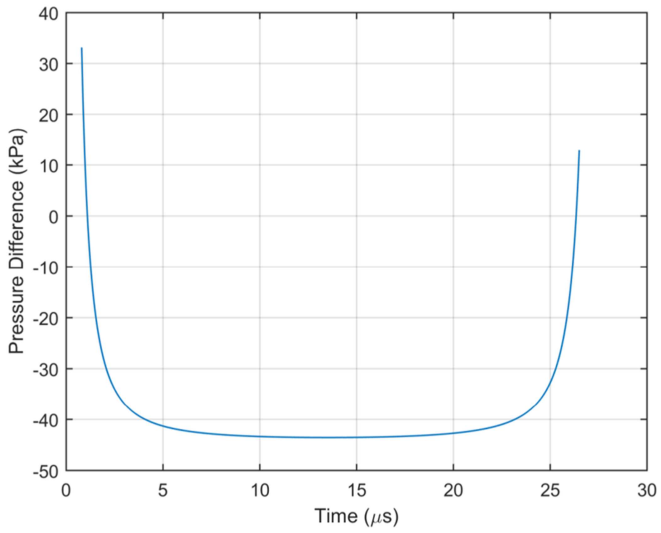

Figure S6 | Pressure difference of the bubble as a function of time. The rapid expansion of the bubble causes the pressure to drop until a negative value is established at about 4 μs. During collapse, the bubble speeds up until the pressure retains a positive value once more, after 23 μs.
